# A Microfluidic Paper-Based Analytical Device for Type-II Pyrethroid Targets in an Environmental Water Sample

**DOI:** 10.3390/s20154107

**Published:** 2020-07-23

**Authors:** Sumate Pengpumkiat, Jintana Nammoonnoy, Watcharaporn Wongsakoonkan, Pajaree Konthonbut, Pornpimol Kongtip

**Affiliations:** 1 Department of Occupational Health and Safety, Faculty of Public Health, Mahidol University, Bangkok 10400, Thailand; pajaree.kon@mahidol.ac.th (P.K.); pornpimol.kon@mahidol.ac.th (P.K.); 2 Chemical Metrology and Biometry Department, National Institute of Metrology (Thailand), Pathumthani 12120, Thailand; jintana@nimt.or.th; 3 Department of Occupational Health and Safety, Faculty of Science and Technology, Valaya Alongkorn Rajabhat University Under the Royal Patronage, Pathumthani 13180, Thailand; watcharaporn@vru.ac.th

**Keywords:** type-II pyrethroids, colorimetric assay, microfluidic device

## Abstract

A detection method for type-II pyrethroids in an environmental water sample using a microfluidic paper-based analytical device (µPAD) is reported here. The detection approach is based on the formation of cyanide from the hydrolysis of type-II pyrethroids and the colorimetric detection of cyanide on a layer-based µPAD. Parafilm and inexpensive laminating pouches were used to create a hydrophobic barrier for the assay on the µPAD. This detection approach was selective to type-II pyrethroids in water for which an environmental water sample was tested. The calibration curves for cypermethrin, deltamethrin, cyhalothrin, and fenvalerate ranged from 2 to 40 µg/mL without sample preconcentration. The lower concentrations of type-II pyrethroids can be assessed by including a preconcentration step prior to the detection on a µPAD. This detection system provides an alternative platform for fast, semiquantitative testing for pesticide contamination in environmental surface water by allowing for portability, low reagent/sample consumption, and low-cost testing.

## 1. Introduction

A microfluidic paper-based analytical device (µPAD) was initially introduced as a point-of-care testing (POCT) device by the Whitesides research group in 2007 [1]. It integrates sample preparation, chemical reaction, and detection in a single step. The device offers several advantages over benchtop laboratory measurements, including high sample throughput, portability, low-cost, low reagent/sample consumption, and disposability. The application of hydrophilic/hydrophobic barriers in the paper substrate allows for effective fluid handling. The µPAD has been applied in healthcare diagnostics [2,3], food quality testing, and environmental monitoring [4,5,6,7]. In terms of pesticide analysis, organophosphate and carbamate can also be analyzed on a µPAD using chemicals and nanoparticles [8,9,10], targeting the inhibition of acetylcholinesterase (AChE) activity by the pesticides for the detection principle [11,12,13,14,15,16,17,18,19].

Fabrication techniques for paper-based devices have been extensively reviewed [20,21] including photolithography, wax printing, ink-jet printing, ink stamping, and plasma treatment. The device created in this study utilized stacking cut pieces of paper as the layers for vertical flow [7,22]. Capillary action and gravitational force facilitate the even flow of the fluid and minimize the channeling effect of the fluid flow. The hydrophobic barrier was made by impregnating the cut paper in a solution of parafilm and toluene. The paper was then allowed to dry to create the finished product, eliminating the need for wax printing in this fabrication model.

**Figure 1:**
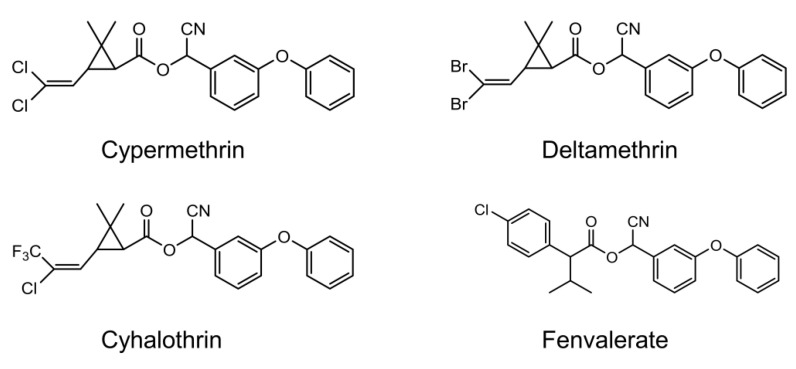
Chemical structure of type-II pyrethroids as target analytes in this study.

Pyrethroids are analogs of natural pyrethrins, which are typically derived from chrysanthemum flowers (*Chrysanthemum cinerariaefolium and C. coccineum*). The structure of synthetic pyrethroids has been chemically modified to provide enhanced toxicity, greater photostability, and selectivity for target species. They are widely used in the cultivation of crops and as effective broad-spectrum insecticides for pest control in urban environments. Pyrethroids are classified into two main categories according to the chemical compositions in their structures. Pyrethroids lacking a cyano group at the α-position are classified as type-I pyrethroids and those with the cyano group are classified as type-II pyrethroids. The most commonly used type-II pyrethroids are deltametrin, cypermethrin, cyhalothrin, acrinathrin, fenpropathrin, β-cyfluthrin, fenvalerate, esfenvalerate, and fluvalinate (Figure 1).

The expansion of monoculture has resulted in the high demand for pesticide usage seen today. Annual imports of pesticides have significantly increased especially in Southeast Asia [23,24]. In 2019, cypermethrin was one of the top three most commonly imported insecticides in Thailand [25]. The potential for inadvertent exposure to pesticides is through agricultural runoff which contaminates the hydrologic system. Once pesticide-contaminated bodies of water reach streams, they can be widely dispersed into rivers, lakes, reservoirs, and oceans [26]. Natural water resources are the most common source for spreading the compounds into the environment and food chains. Although pyrethroids are relatively less toxic to mammals and birds, they are extremely lethal to many aquatic animals and invertebrates [27,28,29,30,31]. In humans, unspecific symptoms are shown after high exposure to pyrethroids, such as faintness, headache, nausea, paresthesia, and respiratory symptoms including cough [32]. Long term exposure may damage the liver by disrupting vital metabolic processes. Pyrethroids act by blocking a target site, sodium channels, and altering the function of gamma-aminobutyric acid (GABA) receptors in nerve filaments [33].

At present, there is no maximum acceptable limit for pyrethroids in surface water quality standards in Thailand [34]. The US Environmental Protection Agency (US EPA) has developed national recommended water quality criteria under Section 304(a) of the clean water act for human health and aquatic life; however, pyrethroids are not included in the list either [35]. Recently, the concentrations and detection rates of pyrethroids in various environmental media have been reviewed and their concentrations in environmental water samples widely expanded ranging from the ng/L level to 13 mg/L [36].

The conventional methods for pyrethroid analysis are gas chromatography-electron capture detector (GC-ECD) [37,38,39,40], gas chromatography–mass spectrometry (GC-MS) [41,42,43,44], liquid chromatography-UV (LC-UV) [45,46], and liquid chromatography–mass spectrometry (LC-MS) [47,48,49,50,51]. The instrumental techniques mentioned above offer high accuracy and precision, good sensitivity, and a very low detection limit, although they are expensive, complicated, and laborious. Thus, these gold standard methods may not be suitable for rapid screening and the detection of pyrethroids in some developing countries which have a low-resource setting but high risk of pesticide exposure.

**Figure 2:**
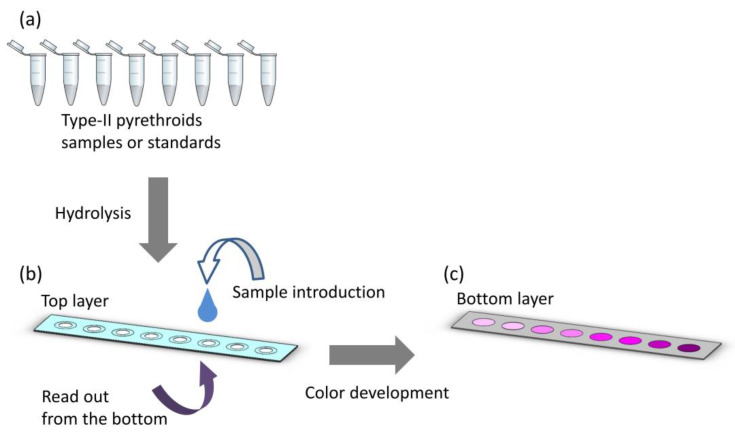
Schematic diagram of the type-II pyrethroid detection method. (**a**) Samples or standards of type-II pyrethroids in microcentrifuge tubes for a hydrolysis reaction. (**b**) The degradation product, cyanide ion testing on the microfluidic paper-based analytical device (µPAD). (**c**) Color development on the µPAD proportional to the type-II pyrethroid concentration.

In this work, we describe our method for creating a layered, paper-based device for type-II pyrethroid screening in an environmental water sample. Type-II pyrethroid pesticides, which contain the cyano group at the α-carbon were hydrolyzed in a solution-based assay, leading to the release of the degradation product, cyanide ions. The cyanide ions were then detected on a µPAD by reacting with ninhydrin (2,2-dihydroxy-1,3-indanedione) to form a colored complex. The color intensity developing on the µPAD was quantitatively measured corresponding to the pyrethroid concentration. Digital image analysis with a red green blue (RGB) color system was used to determine the pyrethroid concentration. The schematic diagram of the method is shown in Figure 2. To the best of our knowledge, our device is the first colorimetric chemosensor for type-II pyrethroid detection on a paper-based device platform.

## 2. Materials and Methods

### 2.1. Chemicals and Materials

All of the type-II pyrethroid standards (Pestanal grade), namely cypermethrin, deltamethrin, cyhalothrin, and fenvalerate were purchased from Sigma-Aldrich (St. Louis, MO, USA). Ninhydrin and polyvinylpyrrolidone (PVP) with an average molecular weight of 10,000 were supplied from Sigma-Aldrich (St. Louis, MO, USA). Ammonium acetate, sodium hydroxide, methanol, ethanol, Triton X-100, dichloromethane, and toluene were obtained from Merck (Darmstadt, Germany). Chromatography paper grade 1CHR was purchased from GE Healthcare (Pittsburgh, PA, USA). Parafilm was purchased from Bemis Company (Oshkosh, WI, USA). Laminating pouch film was purchased from DHA Siamwalla (Bangkok, Thailand).

### 2.2. Optimization of a Hydrolysis Solution for Type-II Pyrethroids

Pyrethroids were hydrolyzed under basic conditions and to yield cyanide ions as a hydrolysis product. The cyanide concentration was then measured using a microplate reader. A hydrolysis solution was designed to contain a polar organic solvent and sodium hydroxide in a ratio of 1:1 [52]. There were two parameters to be optimized, namely the sodium hydroxide concentration (0.025–0.5 M) and types of organic solvent (methanol, ethanol). Cypermethrin at a concentration of 100 µg/mL was used throughout this section as a representative of the pyrethroids. The cypermethrin solutions were mixed thoroughly with a different composition of the hydrolysis solutions in a ratio of 1:1 for 5 min. Twenty microliters of the mixture were transferred into each well of a 96-well plate. One hundred microliters of 4% of ammonium acetate and 1% ninhydrin were added to each well. The 96-well plate was analyzed for absorbance at 570 nm (Synergy HT microplate reader, BioTek, Winooski, VT, USA).

### 2.3. Reagent Preparation for the µPAD

Chromatography paper grade 1CHR was cut into a circle using a hole-punch (6.5 mm diameter) and used as a reagent pad. This µPAD was composed of two layers, including a PVP/buffer layer and sensing layer. The reagents were prepared, spotted on the reagent pads and allowed to air dry in a desiccator at room temperature. To prepare the PVP/buffer layer, 5 µL of 5% polyvinylpyrrolidone (PVP) in 10 mM phosphate buffer pH 7.0 was spotted on the reagent pad. This layer was used for conditioning the sample when the sample passed through the pad layer. PVP is a hydrosoluble polymer to reduce the coffee ring effect of the detection pad [53].

The sensing layer was prepared by spotting with 4 µL of 4% ninhydrin in ethanol on another reagent pad. This layer served as a detection zone. All solutions were kept at 4 °C and stable over a month.

### 2.4. Design of the µPAD

**Figure 3:**
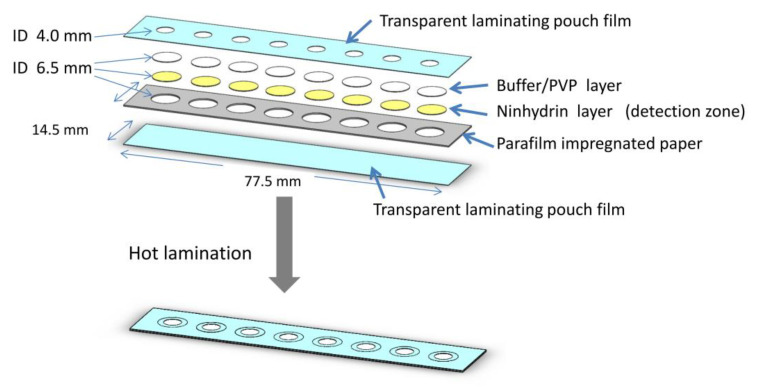
Exploded view of the paper-based analytical device. The dimensions of the paper-based device are 77.5 × 14.5 mm with a channel diameter of 6.5 mm.

A microfluidic paper-based analytical device was designed using SolidWorks 2013 (Dassault Systèmes, Waltham, MA, USA) shown in Figure 3. The device was divided into layers with the detection reagent on the bottom layer. The fluid vertically transported from the top to the bottom layer once applied. The circle channels were cut by a cutting plotter (Silhouette, Lindon, UT, USA) with a diameter of 6.5 mm matching with the reagent pad. Eight individual channels for eight different assays were arrayed in a paper-based device to increase the sample throughput. This µPAD was designed with multiple channels to perform several tests simultaneously including the blank sample.

The device was composed of 3 layers; the top and the bottom layer were transparent laminating pouches. The middle layer was made of white office paper (180 g). Each assay was confined in a hydrophobic barrier by impregnating the cut middle layer of paper in a solution of 5% parafilm in toluene and letting the layer dry at room temperature. All the layers and reagent pads were aligned and assembled using a low-cost thermal laminator. The sample solutions were applied to the top of each channel. The developing color can be photographically measured on the bottom of the transparent pouch on the backside of the paper-based device.

### 2.5. Assay Procedure for Type-II Pyrethroids

Either an environmental water sample or type-II pyrethroid standards were hydrolyzed to generate a stoichiometric degradation product (cyanide ions) in the solution. First, 100 µL of the water sample or standard solution was mixed with 100 µL of a hydrolysis solution containing 50% methanol in 0.25 M NaOH. The mixture was then incubated for 20 min at room temperature. After the reaction was completed, 100 µL of 10% ammonium acetate was added into this mixture. A subsample (5 µL) of the mixture was then applied on a µPAD. The µPAD was allowed to develop the color at room temperature for 6 min prior to the digital image analysis.

### 2.6. Environmental Water Sample with Preconcentration Step

While environmental water samples can be directly analyzed following the assay procedure as previously described, some environmental samples may contain very low concentrations of pyrethroids, below the detection limits of this device without preconcentration. To account for this, environmental water samples can also be preconcentrated via micro liquid–liquid extraction [54,55] and quantified using the assay procedure. To test this, ten milliliters of an environmental water sample was transferred in a 50 mL centrifuge tube and extracted with 1 mL of dichloromethane (DCM) three times. The combined dichloromethane was collected in a vial and gently dried on a heating block at 40 °C. The residue was reconstituted in 100 µL of methanol and tested following the assay procedure in Section 2.5.

### 2.7. Digital Image Analysis and Data Processing

The change in color on the reagent pad was quantitatively proportional to the pyrethroid concentration. An iPhone SE (Model MLXL2LL/A, Apple, Cupertino, CA, USA) was used to take a picture of a µPAD under a controlled-light box. Pictures of the controlled-light box and the specifications of the light are shown in the Electronic Supplementary Materials (Figure S1). The light box helped maintain consistent light intensity for photo taking and prevented stray light or user’s shadow from interfering with the result. Furthermore, if used in the field, the lightbox would eliminate interference from sunlight. RGB color intensities on the reagent pad were obtained using ImageJ (version 1.50b, NIH, USA). In the detection zone, cyanide ions derived from the pyrethroid target form a purple complex with ninhydrin. First, an image of the whole µPAD was taken. A circular area of the uniform color on the detection zone for each individual well was selected one at a time and measured by selecting the “Measure RGB” function to obtain average RGB color intensities. The raw RGB signals decrease as the pyrethroid concentration increases, which initially gives a negative slope for calibration. In order to construct a positive slope, which is favorable for quantitative analysis, absolute color intensity or ΔRGB was calculated. The control or blank sample was used as a calibrator to calculate the absolute color intensity as follow [56,57];

(1) $$\mathrm{Color}\;\mathrm{intensity}\;=\sqrt{{\left(R-R_{B}\right)}^{2}+{\left(G-G_{B}\right)}^{2}+{\left(B-B_{B}\right)}^{2}}$$

*R* is the red intensity of the sample, $R_{B}$ is the red intensity of the blank, *G* is the green intensity of the sample, $G_{B}$ is the green intensity of the blank, *B* is the blue intensity of the sample, $B_{B}$ is the blue intensity of the blank. ΔRGB is advantageous as there is evidence of less variability in measurements between smartphone types and less chance that a small error in a single RGB channel would impact results [58]. By plotting the absolute color intensities with the increasing concentrations of the pyrethroids, a positive slope of the calibration curves was obtained and facilitated its application in quantitative analysis.

## 3. Results and Discussion

### 3.1. Hydrolysis of Cypermethrin

The detection principle of this work was based on the hydrolysis of type-II pyrethroid pesticides to release cyanide ions in solution and further quantitatively determine the cyanide concentration on the µPAD. Under alkaline conditions, pyrethroid hydrolysis can occur as shown in Figure S2 [52]. The hydrolysis mechanism of type-II pyrethroids is similar to the mechanism of simple aliphatic esters. The final products include the organic acid (RCOO^-^), 3-phenoxybenzaldehyde (3-PBA), and cyanide.

**Figure 4:**
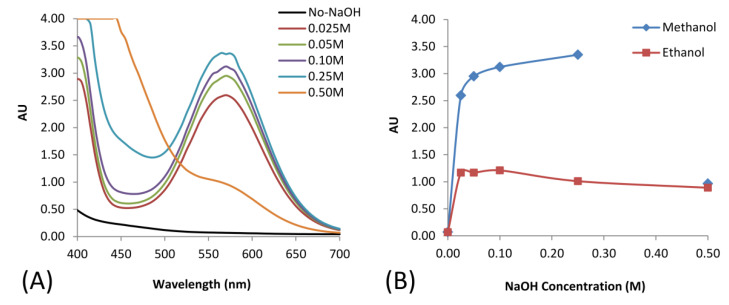
(**A**) Scanning absorbance spectra of the resulting solutions derived from using different concentrations of NaOH mixed with methanol in a ratio of 1:1 as a hydrolysis solution (after 15 min of developing reactions); (**B**) The effect of organic solvent with the different concentrations of NaOH vs the absorbance at a wavelength of 570 nm.

In this study, we firstly examined the pyrethroid hydrolysis condition in a solution-based assay. Sodium hydroxide at different concentrations from 0.025 to 0.5 M in different polar organic solvents was studied. The organic solvent helped increase the solubility of the pyrethroids in an aqueous solution which contained the hydroxyl ion and allowed the hydrolysis to take place. The resulting cyanide further reacted with ninhydrin and ammonium acetate in a solution-based assay forming a colored complex. The reactions were monitored using a UV–Vis spectrophotometer. The results are shown in Figure 4.

The effect of sodium hydroxide concentration on hydrolysis is displayed in Figure 4A. The results indicated that the pyrethroid hydrolysis increased with the increase of sodium hydroxide concentration. The effect of the polar organic solvent is illustrated in Figure 4B, which shows that methanol gave a higher yield than ethanol. Therefore, to obtain the maximum hydrolysis yield, 0.25 M of sodium hydroxide and methanol at a volume ratio of 1:1 *v/v* were used for further studies.

### 3.2. Detection Mechanism of the Type-II Pyrethroid Targets

**Figure 5:**
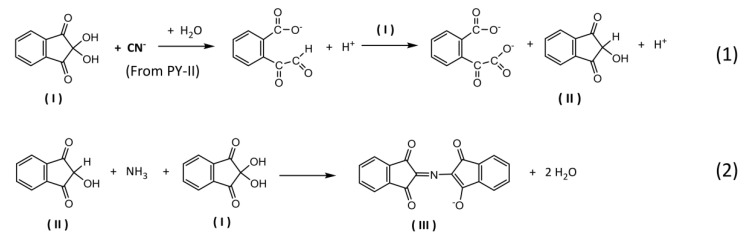
Schematic reaction of ninhydrin with type-II pyrethroid hydrolysis product on a µPAD format. Cyanide reacts with ninhydrin (I) to give 2-hydroxy-1,3-indanedione (II) (1), followed by the formation of Ruhemann’s purple (III) (2).

The analytical colorimetric assay for a µPAD was based on the chemical reaction of ninhydrin, a chromogenic agent corresponding to the pyrethroid hydrolysis product, cyanide. It is generally known that ninhydrin is a common reagent for qualitative assay for α-amino acids [59,60] by oxidative deamination and decarboxylation of amino acid to produce a purple-colored product. Interestingly, the reaction mechanism of ninhydrin considerably varies among organic chemistry and biochemistry contexts [59]. Recently, ninhydrin has been applied to quantitatively determine free cyanide in a medium of sodium carbonate [61,62,63]. In this work, we focused on the reaction mechanism of ninhydrin to cyanide with some modifications facilitating the use of dry reagents on a µPAD platform. The overall schematic equation for the modified condition is proposed as shown in Figure 5. The pyrethroid hydrolysis product, cyanide, works as a selective reducing agent for ninhydrin to form 2-hydroxy-1,3-indanedione (II). It later couples with another molecule of ninhydrin and a free ammonium ion resulting in diketohydrindylidene-diketohydrindamine or Ruhemann’s purple (III). The color intensity of Ruhemann’s purple stoichiometrically corresponds to the pyrethroid concentration.

### 3.3. Assay Optimization and Measurement of Type-II Pyrethroids

The parameters affecting the analytical signal of the pyrethroids were optimized to enhance the sensitivity of the assay on a µPAD, including ninhydrin concentration, ammonium acetate concentration, and reaction time for colorimetric assay. These parameters directly affect the chemical reactions for the formation of Ruhemann’s purple. 

**Figure 6:**
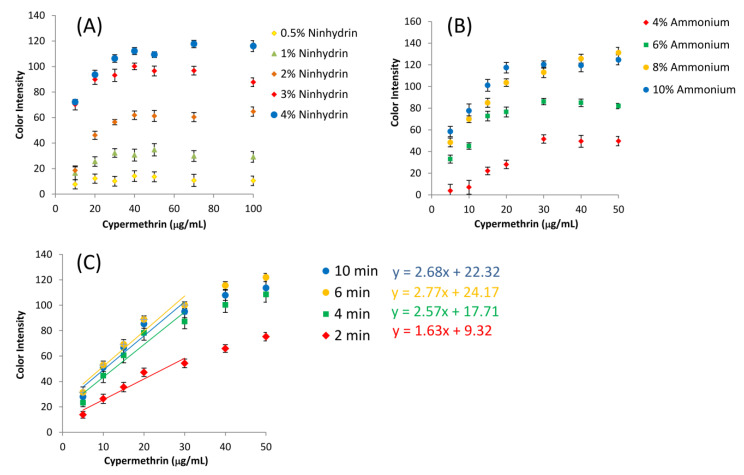
Optimization of the assay on a µPAD for different parameters: (**A**) ninhydrin concentrations; (**B**) ammonium acetate concentrations; (**C**) assay color intensities plotting against cypermethrin concentrations at different developing times (n = 3), the error bars represents the standard deviation.

Ninhydrin was used as the chromogenic agent for the assay. It was predeposited on a 1CHR paper dot on the bottom of a µPAD. Different ninhydrin concentrations were tested to determine the effect on the color intensity of the assay. The concentration varied from 0.5% to 4% due to the fact that at a concentration higher than 4%, ninhydrin cannot completely dissolve in ethanol and precipitates. The relationship between the concentrations of cypermethrin and ninhydrin is shown in Figure 6A. The slope of the calibration graph dramatically increased from 1% to 4% ninhydrin. At the concentration of 0.5% of ninhydrin, there was no observable signal from the assay. The color intensity was proportional to the concentration of cypermethrin and saturated after the cypermethrin concentration reached 40 µg/mL. Therefore, the ninhydrin concentration was set at 4% for further experiments.

To generate the ninhydrin chromophore, ammonium acetate was added in the solution-based assay prior to applying on a µPAD. It contains ammonia which is another one of the reactants to form the final product, Ruhemann’s purple. The ammonium acetate concentration varied from 2% to 10% with a fixed volume of 1:1 ratio to the sample. As the concentration of ammonium acetate increased (4–10%), the slope of the calibration curve increased and reached the constant at 10% as shown in Figure 6B. A concentration lower than 2% showed no signal of the assay product and no relationship between the cypermethrin concentration and the color intensity (data not shown). Therefore, 10% of ammonium acetate was chosen as the condition for further studies.

The reaction time was the time started from the introduction of the premixed standards/samples on a µPAD until the color measurement. The color development started instantly after the solution flowed and reached the µPAD. The effects of reaction time were also observed as shown in Figure 6C. The slope of calibration indicated the sensitivity of the detection mechanism. As the reaction time increased, the slope also increased from 1.63 to 2.77. After 6 min, the assay reached the endpoint of the reaction. Therefore, the optimized reaction time was set at 6 min to compromise between higher sensitivity, a wider range of concentration, and shorter analysis time.

### 3.4. Assay Selectivity for Type-II Pyrethroids

**Figure 7:**
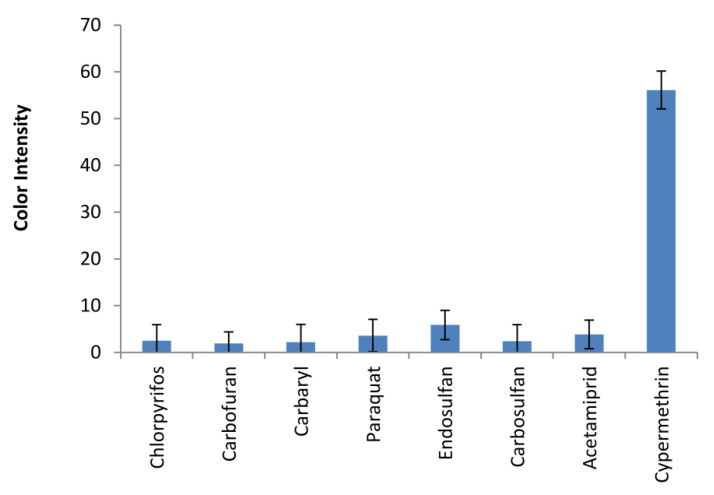
The assay selectivity of a microfluidic paper-based device for type-II pyrethroids. Several pesticides generally used in agricultural fields were tested with a µPAD compared to the signal of cypermethrin at the concentration of 10 µg/mL (chlorpyrifos 16 µg/mL, carbofuran 1097 µg/mL, carbaryl 1202 µg/mL, paraquat 2000 µg/mL, endosulfan 51 µg/mL, carbosulfan 1000 µg/mL, acetamiprid 1000 µg/mL).

The selectivity of the assay on a µPAD was evaluated against a wide variety of pesticides used in the agricultural fields namely chlorpyrifos, carbofuran, carbaryl, endosulfan, carbosulfan, and acetamiprid. The responses as color intensities of various pesticides were observed as shown in Figure 7. Cypermethrin gave the highest color intensities among other pesticides; however, high concentrations of other pesticides generated low background signals for the assay. Therefore, this paper-based analytical device with a premixed hydrolysis solution on the ninhydrin assay has proven to be selective to type-II pyrethroids.

The drawback of all chemical sensors is the possibility of cross-reactivity. Based on the reagent used on the assay and mechanism of the detection, free amino acids and free cyanide are potential interferences that cause false-positive signals for pyrethroid targets. For those samples that have the interferences, a sample cleanup/preconcentration, such as C18-solid phase extraction could be used before detection on a device to eliminate the false positives.

### 3.5. Calibration Graph of Type-II Pyrethroid Analysis

**Figure 8:**
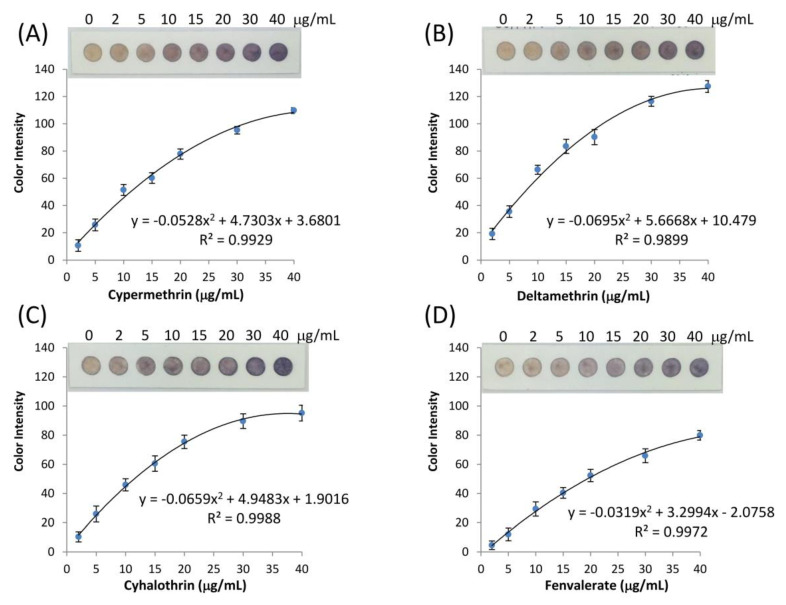
The calibration curves for type-II pyrethroids detection along with the visual µPAD, (**A**) cypermethrin, (**B**) deltamethrin, (**C**) cyhalothrin, and (**D**) fenvalerate at optimum conditions (n = 5).

The performance of a microfluidic paper-based analytical device was evaluated for type-II pyrethroids in an environmental water sample. Deionized water was used initially for method development. The pyrethroid type-II standards including cypermethrin, deltamethrin, cyhalothrin, and fenvalerate were used. A series of concentrations were prepared in the range of 0–40 µg/mL and analyzed following the assay procedure. The analytical signal from the assay was the color change from white to purple, characterized by the RGB color intensity in digital image analysis. Traditionally, if the RGB color intensities were plotted against the concentration, the slope of the calibration would be negative because a white color gives each red, green, and blue values of 255, while the darker color gave less RGB values. In this work, an absolute color intensity presented here is a combination of red, green, and blue channels of the test assay compared to the signals from the blank [56,57]. This plot resulted in a positive slope for the calibration graph which is favorable and suitable for quantitative chemical analysis. The calibration graphs between absolute color intensity and the pyrethroid concentration on the µPAD are presented in Figure 8. The regression lines were fitted by a two-degree polynomial equation in a range from 2 to 40 µg/mL (overall R^2^ > 0.98). The linear correlations were obtained at the lower concentration range from 2 to 15 µg/mL shown in Figure S3. Limits of detection of cypermethrin, deltamethrin, cyhalothrin, and fenvalerate were determined following Holstein et al. [64] and reported as 2.50, 1.06, 3.20, and 5.73 µg/mL, respectively. This calculation approach contemplates the standard deviation of the blank samples and the test samples from the calibration graphs. The calculation table was shown in Electronic Supplementary Materials (Table S1). The color intensity changes due to the presence of pyrethroids shown in Figure 8, can also be observed by naked eyes, which make it feasible for semiquantitative analysis on a color chart.

### 3.6. Measurement of Type-II pyrethroid Targets in an Environmental Water Sample

We demonstrated the performance of type-II pyrethroid detection on a µPAD without sample preconcentration earlier. The feasibility to trace the lower concentration of pesticides in some water supplies was also studied by adding the preconcentration step before applying to the assay. For an environmental water sample, surface water from NakhonNayok province, Thailand was collected, filtered, and used as a blank sample. Standard pesticides of type-II pyrethroids were fortified in the blank sample. The fortified samples were preconcentrated 100 times by taking 10 mL of the sample and performing liquid–liquid extraction × 3 times with dichloromethane. The final volume of the extract for a µPAD assay was 100 µL [55]. The accuracy and precision of the type-II pyrethroid detection on a µPAD were expressed as a percentage of recovery and the standard deviation from individual µPAD. The data shown in Table 1 were calculated and obtained from the linear calibration method.

The recoveries of deltamethrin, cyhalothrin, and fenvalerate ranged from 83.73% to 106%, which were in the acceptable range proposed by AOAC [65] Cypermethrin at the concentration of 0.10 µg/mL gave 78.31% recovery, which was slightly lower than the acceptable range. The large standard deviations shown in this section largely resulted from the sample preconcentration step prior to the detection of type-II pyrethroids on a µPAD.

## 4. Conclusions

We have successfully developed a paper-based analytical device for detecting type-II pyrethroid pesticides in a liquid sample. Cyano moieties, which are the unique characteristic side chains of the type-II pyrethroid molecules, were initially hydrolyzed in a solution. The optimized solution for hydrolysis was 50% methanol in 0.25 M NaOH at room temperature. Free cyanide ions as the resulting degradation product in the mixture were later detected on a µPAD using a ninhydrin assay. Color intensities of Ruhemann’s purple quickly developed and stoichiometrically correlated to the pyrethroid concentration in a range of 2–40 µg/mL. The detection limit of cypermethrin, deltamethrin, cyhalothrin, and fenvalerate was reported as 2.50, 1.06, 3.20, and 5.73 µg/mL, respectively. The lower concentration of type-II pyrethroids in some surface water samples can be assessed and quantitated by adding a sample preconcentration step before the assay procedure as outlined in this paper. The layered-based design of the µPAD allowed for the simple and easy fabrication of this device. The parafilm impregnated paper used in this design proved to be an effective hydrophobic barrier for each individual assay. This design allowed the fluid to descend vertically and homogeneously to the bottom layer for detection. This µPAD has the potential to fulfill the demand for a reliable, semiquantitative screening tool for type-II pyrethroids at ppm levels and sub-ppm levels with a sample preconcentration step.

## Figures and Tables

**Table 1:** Recovery test of a microfluidic paper-based device for type-II pyrethroids with sample preconcentration from an environmental water sample (n = 3).

Analyte	Added Concentration	Found Value	Recovery
	(µg/mL)	(µg/mL) ± SD	(%) ± SD
Cypermethrin	0.10	0.078 ± 0.013	78.31 ± 13.14
	0.15	0.141 ± 0.026	93.74 ± 17.11
Deltamethrin	0.10	0.086 ± 0.045	86.27 ± 44.66
	0.15	0.131 ± 0.037	87.14 ± 24.83
Cyhalothrin	0.10	0.084 ± 0.029	83.73 ± 28.93
	0.15	0.137 ± 0.032	91.07 ± 21.10
Fenvalerate	0.10	0.085 ± 0.017	84.55 ± 17.02
	0.15	0.159 ± 0.022	106.01 ± 14.44

## Supplementary Materials

The following are available online at https://www.mdpi.com/1424-8220/20/15/4107/s1, Figure S1: The pictures of a controlled-light box and the specifications of the light, Figure S2: Schematic diagram of the hydrolysis reaction of Type-II pyrethroids, Figure S3: Linear calibration graph of type-II pyrethroids in a range of 2–15 µg/mL. (A) cypermethrin, (B) deltamethrin, (C) cyhalothrin, (D) fenvalerate., Table S1: Limit of detection analysis for type-II pyrethroids.
