# Supplementary material for: A Microfluidic Paper-Based Analytical Device for Type-II Pyrethroid Targets in an Environmental Water Sample
Source: Sensors (Basel). 2020 Jul 23;20(15):4107. doi: 10.3390/s20154107 (PMC7435633; doi:10.3390/s20154107)
Supplement: Supplementary file 1 [file sensors-20-04107-s001.pdf]

Supplementary materials

A Microfluidic Paper-Based Analytical Device for Type-II Pyrethroid Targets in an Environmental Water Sample

Sumate Pengpumkiat [1,]*, Jintana Nammoonnoy [2], Watcharaporn Wongsakoonkan [3], Pajaree Konthonbut [1] and Pornpimol Kongtip [1]

[1] Department of Occupational Health and Safety, Faculty of Public Health, Mahidol University, Bangkok 10400, Thailand; pajaree.kon@mahidol.ac.th (Pa.K.); pornpimol.kon@mahidol.ac.th (Po.K.)
[2] Chemical Metrology and Biometry Department, National Institute of Metrology (Thailand), Pathumthani 12120, Thailand; jintana@nimt.or.th
[3] Department of Occupational Health and Safety, Faculty of Science and Technology, Valaya Alongkorn Rajabhat University Under the Royal Patronage, Pathumthani 13180, Thailand; watcharaporn@vru.ac.th
* Correspondence: sumate.pen@mahidol.ac.th; Tel.: +6696-891-9531

Table of content — Page

Figure S1. The pictures of a controlled-light box — 2

Figure S2. Schematic diagram of the hydrolysis reaction of type-II pyrethroids — 3

Figure S3. Linear calibration graph of type-II pyrethroids — 4

Table S1. Limit of detection analysis for type-II pyrethroids — 5

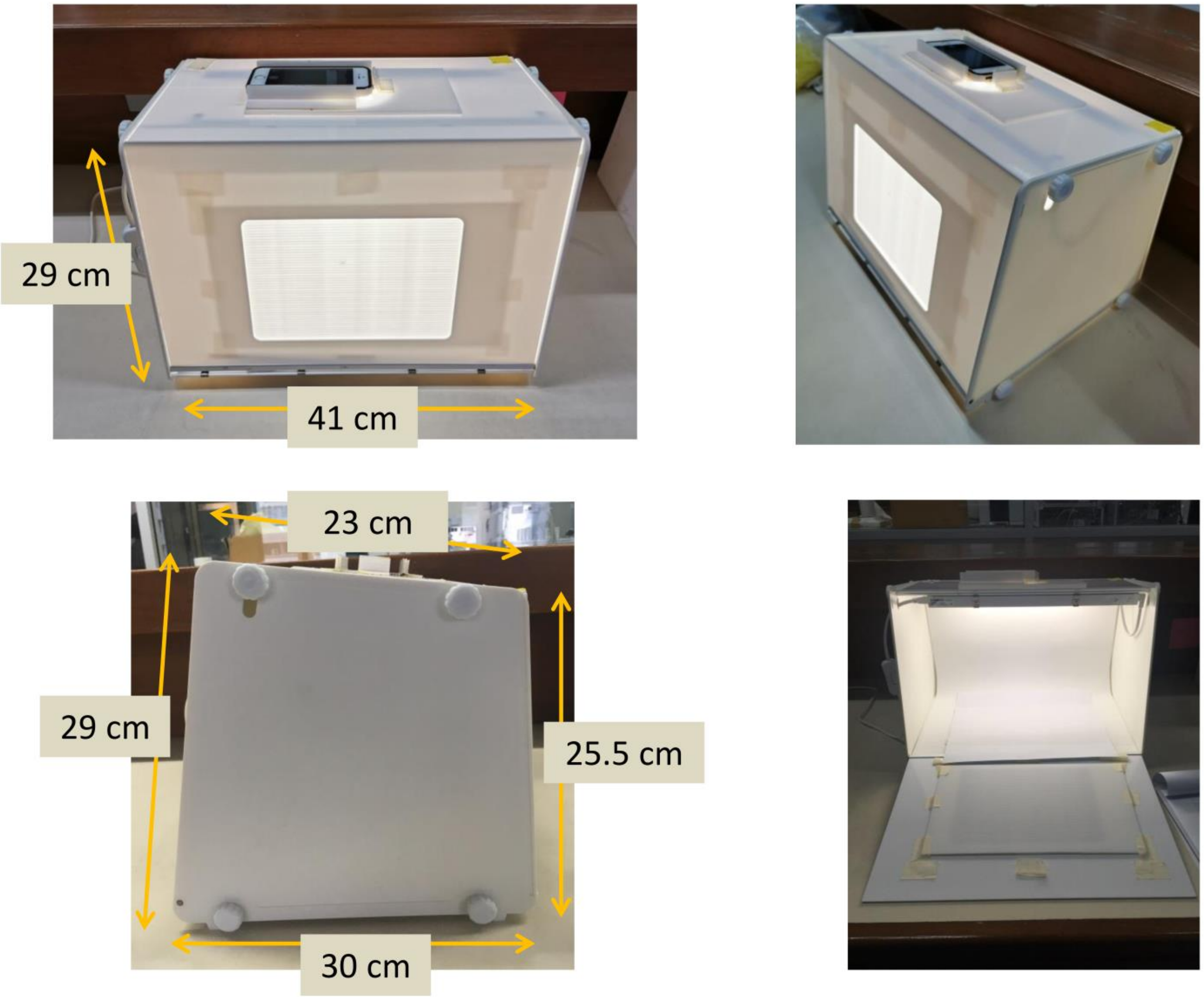

Brand	Sanoto MK40
Box material	ABS + PMMA
Type of light	Fluorescent
Color temperature	5500 K
Voltage	AC 220V
Frequency	50-60 Hz
Distance between smartphone and the device	21 cm

Figure S1. The pictures of a controlled-light box and the specifications of the light.

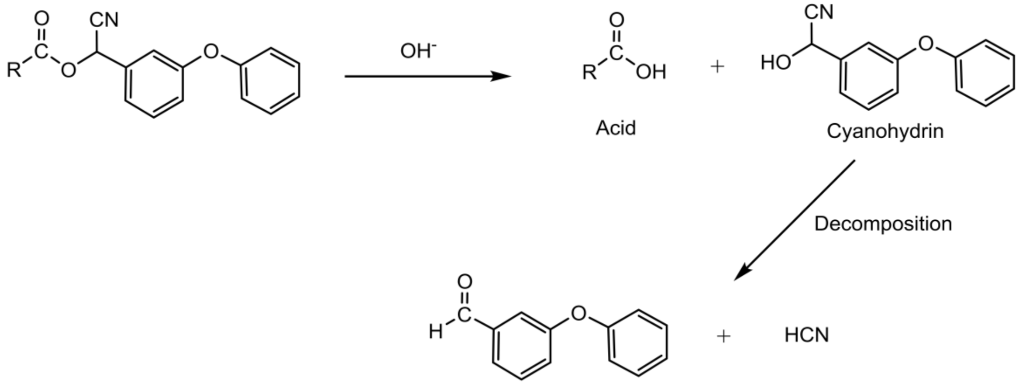

Name	Alkyl group (R-)	Name	Alkyl group (R-)
Cypermethrin	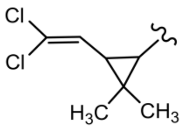	Cyhalothrin	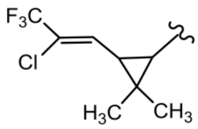
Deltamethrin	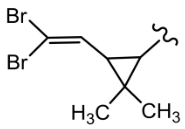	Fenvalerate	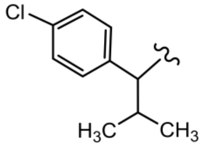

Figure S2. Schematic diagram of the hydrolysis reaction of Type-II pyrethroids.

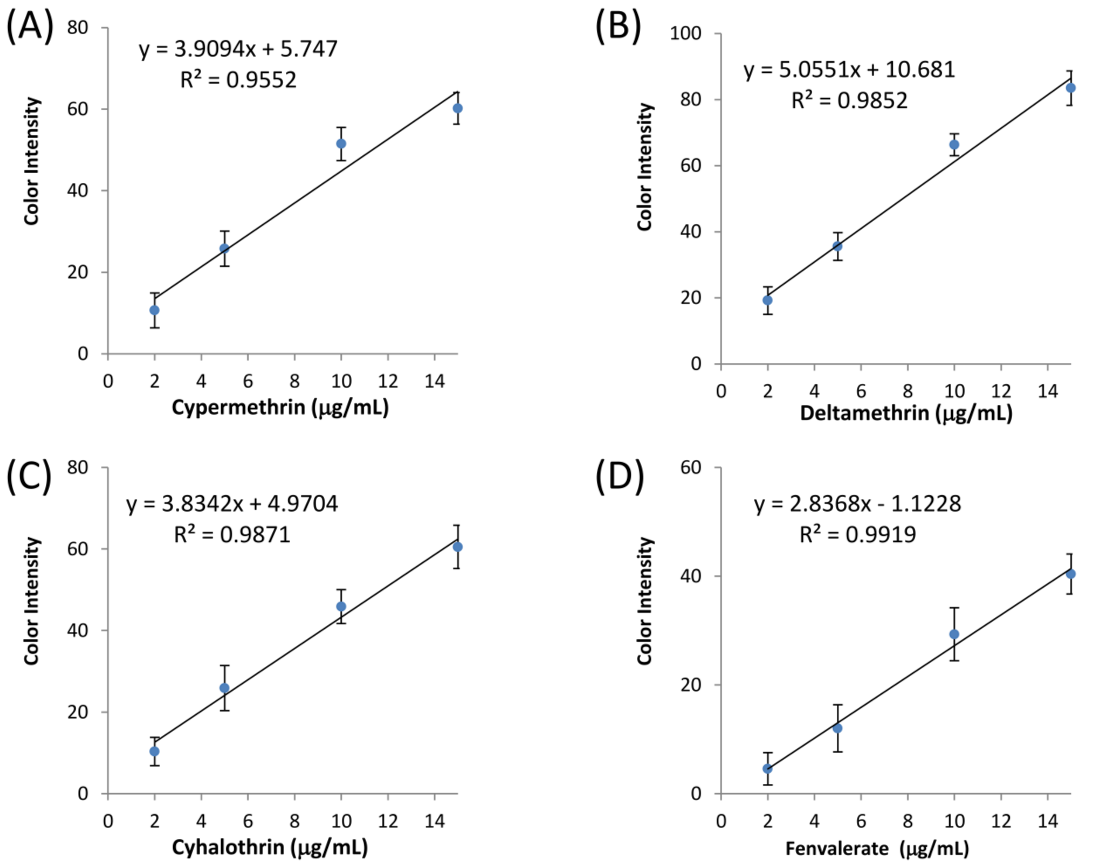

Figure S3. Linear calibration graph of type-II pyrethroids in a range of 2 - 15 μg/mL. (A) Cypermethrin, (B) Deltamethrin, (C) Cyhalothrin, (D) Fenvalerate.

Table S1. Limit of detection analysis for type-II pyrethroids

Step	Value	Result				domain
		Cypermetrhin	Deltamethrin	Cyhalothrin	Fenvalerate	
1	L_C	9.15	9.15	9.15	9.15	signal
2	σ_{test}	3.67	4.41	4.83	4.06	signal
3	L_D	15.17	16.38	17.08	15.81	signal
4	Calibration curve	$Y = -0.0528x^2 + 4.7303x + 3.6801$	$Y = -0.0695x^2 + 5.6668x + 10.479$	$Y = -0.0659x^2 + 4.9483x + 1.9016$	$Y = -0.0319x^2 + 3.2994x - 2.0758$	Signal-to-concentration
5	LOD	2.50	1.06	3.20	5.73	concentration (μg/mL)
